# Reducing expression of *TaOTUB1s* decreases tiller number in wheat

**DOI:** 10.1080/15592324.2021.2018217

**Published:** 2021-12-30

**Authors:** Deng Ji Jiang, Xiao Hua Hao, Jing Zhang, Heng Tang, Fang Wang

**Affiliations:** a State Key Laboratory of Crop Biology, College of Life Sciences, Shandong Agricultural University, Taian, Shandong, China; b College of Plant Protection, Shandong Agricultural University, Taian, Shandong, China

**Keywords:** Otubain, *TaOTUB1*s, tiller number, wheat

## Abstract

Tiller number is an important agronomic trait that affects crop yield. The roles of *OTUB1* in regulating tiller numbers in rice have been reported. However, the roles of *OTUB1* in wheat remain elusive. In this study, *TaOTUB1*s were identified in wheat. TaOTUB1 proteins were localized in the nucleus and cytoplasm. Compared with wild-type Fielder, *TaOTUB1*-RNAi transgenic wheat plants had fewer tillers. Similar to *OTUB1* in rice, the yeast double hybrid indicated that the TaOTUB1-A protein could interact with TaSPL17 and TaUBC13 proteins. The results of quantitative real-time polymerase chain reaction revealed that the expression levels of *TaOTUB1*s decreased while those of *TaSPL17* significantly improved in *TaOTUB1*-RNAi lines. These findings suggested that TaOTUB1s influenced tiller number in wheat.

## Introduction

Wheat is an important crop that supplies about 20% of the total food for the world population. Tiller number is an important agronomic trait that may influence the yield by directly affecting the spike number in wheat. Therefore, identifying genes or alleles correlated with tiller number in wheat breeding programs is essential.

Tiller number is regulated by several genetic pathways in wheat. Tiller development includes two steps. First, an axillary meristem is generated in each leaf axil, followed by an outgrowth that develops into an axillary bud. Second, the axillary bud may remain dormant or develop into a branch responding to various hormone signals.^1–3^ To date, only a few genes/loci are found to be associated with tiller number due to the complexity of the wheat genome. Four single genes (*tin1, 2, 3*, and *ftin*) responsible for tiller inhibition have been reported.^4–7^ TaMOC1, a GRAS transcription factor, may regulate the axillary meristem initiation.^8^ *TaD27-B* affects the outgrowth of tiller bud by affecting the strigolactone level in bread wheat. miR156-TaSPLs controls tiller number by interacting with the strigolactone signaling repressor TaD53.^9^

The ubiquitin–proteasome system regulates almost every aspect of growth and development in plants. Protein ubiquitination is a reversible event. Ubiquitin can be added to target proteins by ubiquitination and be cleaved off from ubiquitinated proteins by deubiquitinating enzymes (DUBs).^10^ DUBs, which belongs to the thiol protease group, can be divided into five families according to their structure of the active site and the catalytic mechanism: ubiquitin-specific proteases/processing proteases, ovarian tumor domain–containing proteases (OTUs), Machado–Joseph disease protein domain proteases (MJD), ubiquitin carboxy-terminal (UCH) proteases, and JAB1/MPNC/MOV34 (JAMMs) proteases.^11^

The OTU was first found in *Drosophila melanogaster*, which contains a conserved catalytic triad including aspartate, cysteine, and histidine.^12^ OTUs have been extensively studied in mammals. OTUs contain four subfamilies in humans: the OTU domain–containing ubiquitin aldehyde-binding protein subfamily (Otubains), the ovarian tumor domain–containing proteins (OTUDs) subfamily, the A20-like subfamily, and the OTU DUB with linear linkage specificity (OTULIN).^13^ OTUs may participate in the host–pathogen interaction in eukaryotic cells and are involved in the deubiquitinating activity.^14^ OTUB1 is an extensively studied member of the OTU deubiquitinating family. The human *OTUB1* gene is located on chromosome 11 and is related to DNA damage repair and the occurrence of malignant tumors.^15^ Several OTU genes have been characterized in plants. A total of OTU domain–containing DUBs are present in the Arabidopsis genome.^16^ OTLD1 is an Otubain-like protein that interacts with KDM1C and can limit plant growth by promoting the concerted epigenetic regulation of a set of genes via histone deubiquitination and demethylation.^17^ OTUB1/WTG1 determines grain size and shape in rice.^18^ Besides, OTUB1 is also related to reduced tiller number and thickened culm in rice.^19^ However, our understanding of the functions and molecular mechanisms of OTUs in plants is still limited.

The function of Otubain proteases in wheat are still unclear. In this study, the wheat *TaOTUB1*s were identified through bioinformatics analysis, their subcellular location and expression pattern were examined, and then their function was analyzed through genetic transformation. This study revealed the effect of *TaOTUB1*s on tiller number and provided a theoretical basis for the molecular design breeding of wheat.

## Results and discussion

### Isolation of TaOTUB1s

**Figure 1.**
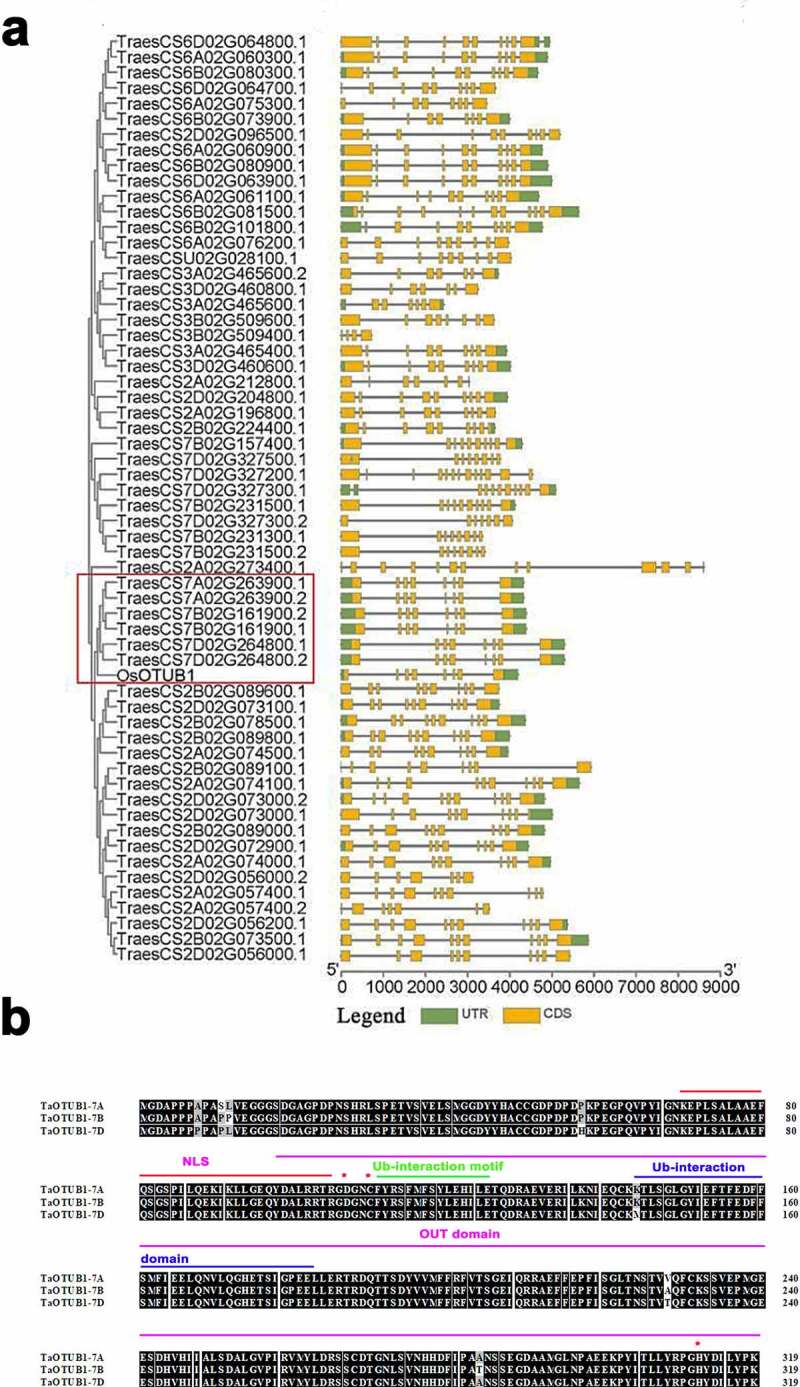
(a) Evolutionary tree and gene structure analysis of the Peptidase_C65 Otubain family in wheat. (b) Protein sequence alignment of TaOTUB1s. The asterisk represents the cysteine triplet catalytic enzyme site.

In this study, wheat Otubain genes were identified using the Hidden Markov Model (HMM) profiles of the Peptidase C65 Otubain domain (Pfam ID: PF10275.2) blasting the wheat genome databases (http://plants.ensembl.org/index.html). A total of 50 sequences were discovered as potentially encoding Otubain proteins in the wheat genome, of which nine genes contained two transcripts. The phylogenetic analysis of the wheat Otubain proteins showed that TaOTUB1-A (TraesCS7A02G263900), TaOTUB1-B (TraesCS7B02G161900), and TaOTUB1-D (TraesCS7D02G264800) proteins were closely related to rice OTUB1(Os08g42540) (Figure 1a).

*TaOTUB1-A, TaOTUB1-B*, and *TaOTUB1-D* encoded putative products comprising 319 amino acids. TaOTUB1-A has extremely high amino acid identities of 98.70% and 98.26% with TaOTUB1-B and TaOTUB1-D, respectively. The sequencing analysis revealed that TaOTUB1s contained OTU domains. TaOTUB1s contained the conserved cysteine, histidine, and aspartate residues that defined the putative catalytic triad of cysteine proteases. Moreover, the Ub-interaction motif and Ub-associated domain were conserved in proteins of the Ub pathway.^20,21^ They were all found in TaOTUB1s. In addition, TaOTUB1s had putative nuclear localization signals (Figure 1b). Based on the aforementioned results, it was confirmed that *TaOTUB1* genes had been identified in hexaploid wheat.

### Subcellular localization and expression patterns of TaOTUB1 proteins

**Figure 2.**
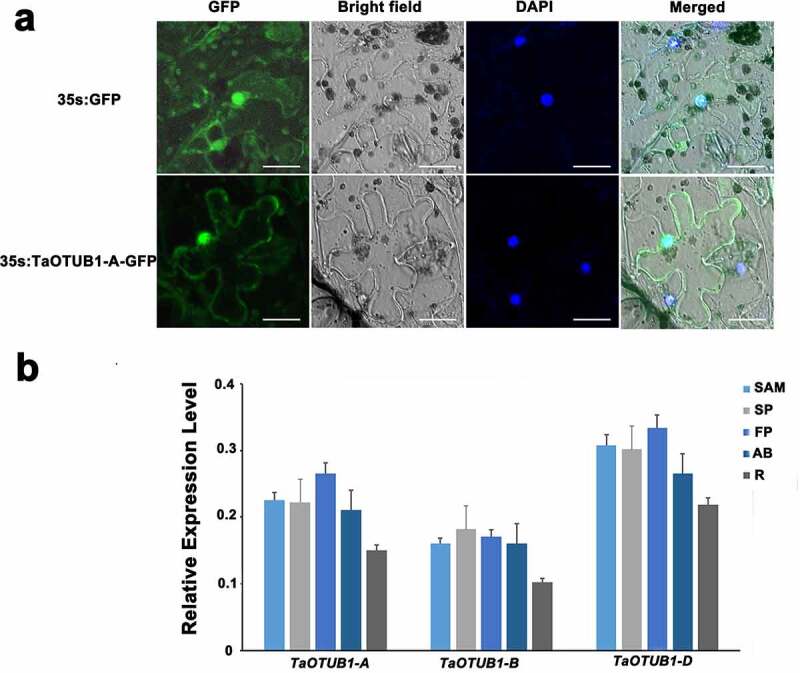
(a) Subcellular localization analysis of TaOTUB1-A protein. Bar = 25 µm. (b) Expression level of TaOTUB1s in the stem apical meristems (SAM) of single-ridge stage , spikelet primordia (SP), floret primordia (FP), axillary buds (AB), and roots (R).

The full-length cDNA of *TaOTUB1-A* was connected to the 35S::GFP vector to construct the 35S::TaOTUB1-GFP fusion expression vector. Using *Agrobacterium* to infect tobacco, the epidermal cells of tobacco leaves were infected with the 35S::TaOTUB1-GFP vector instantaneously. Strong GFP fluorescence signals were detected in the nucleus and cytoplasm (Figure 2a–c). It indicated that the TaOTUB1-A protein was located in the nucleus and cytoplasm. Considering the high similarity among the three TaOTUB1s (>98.0%), we inferred that TaOTUB1s were probably located in the nucleus and cytoplasm.

qPCR was carried out to analyze the expression patterns of TaOTUB1s in different organs and stages of wheat. The results showed that TaOTUB1 homeologs were expressed in all examined organs, including stem apical meristems of the single-ridge stage, spikelet primordia, floret primordia, axillary buds, and roots (Figure 2d). TaOTUB1 homeologs had similar expression patterns, suggesting that they might play similar roles in wheat. *TaOTUB1-A* was chosen as the research object in this study.

### Phenotypic analysis of TaOTUB1-RNAi plants

The conserved region was amplified and subcloned to construct an RNAi vector (*TaOTUB1*-RNAi). The *TaOTUB1*-RNAi vector was transferred into *T. aestivum* cv. Fielder. In total, 16 *TaOTUB1-*RNAi transgenic lines were obtained. Three T2 generation lines (102#, 405#, and 513#) were selected for phenotypic observation.

**Figure 3.**
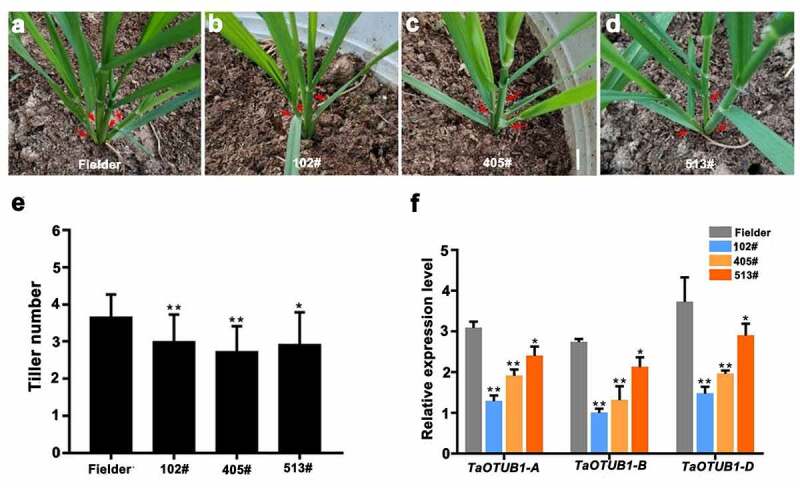
(a–d) Morphological characteristics of wild-type Fielder and *TaOTUB1*-RNAi plants. Red arrows show the positions of tillers in the seedling stage. (e) Statistical analyses of the tiller numbers of the wild-type Fielder and three independent *TaOTUB1*-RNAi lines (*n* = 15, *P < 0.05, **P <0 .01). (f) Expression analysis of *TaOTUB1*s in the wild-type Fielder, 102#, 405#, and 513# plants.

**Figure 4.**
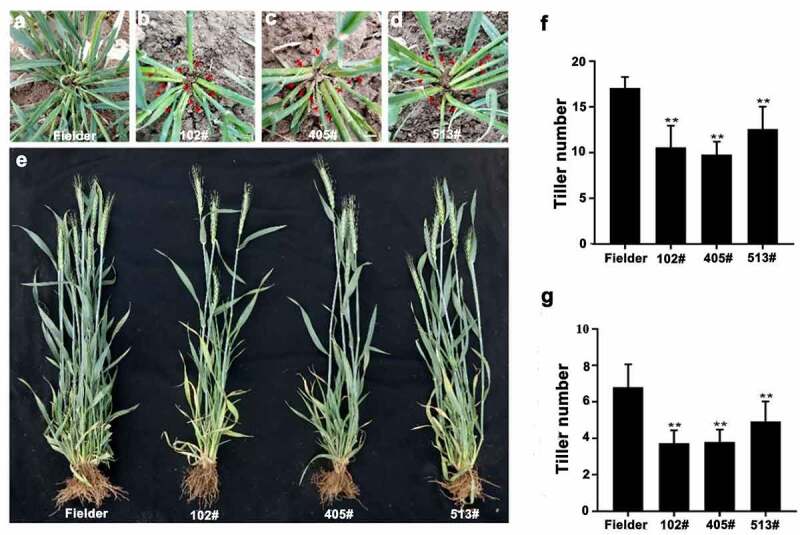
(a–d) Morphological characteristics of wild-type Fielder and T2-generation *TaOTUB1*-RNAi transgenic wheat in the jointing stage. Red arrows indicate the positions of tillers. (e) Morphological characteristics of wild-type Fielder and T2-generation *TaOTUB1*-RNAi transgenic wheat in the heading stage. (f) Statistical analyses of the tiller numbers of the wild-type Fielder and three independent transgenic lines in the jointing stage (*n* = 15, **P <0 .01). (g) Statistical analyses of the tiller numbers of the wild-type Fielder and three independent transgenic lines in the heading stage (*n* = 15, **P <0 .01).

We focused on analyzing the biological function of *TaOTUB1s* in regulating tiller numbers in detail. In the seedling stage, reduced numbers of visible tillers were observed in *TaOTUB1*-RNAi transgenic plants compared with wild-type Fielder plants (Figure 3). Similarly, in the jointing and heading stages, three *TaOTUB1*-RNAi transgenic lines still had fewer tillers compared with the wild-type Fielder plants, with an average reduction of two tillers. The plant type appeared loose in *TaOTUB1*-RNAi transgenic plants compared with the wild-type control (Figure 4). Further, the expression levels of *TaOTUB1*s in transgenic plants and wild-type Fielder were analyzed. The expression levels of *TaOTUB1*s all were significantly reduced in *TaOTUB1*-RNAi transgenic plants (Figure 3f). Thus, the results demonstrated that decreased expression of *TaOTUB1s* suppressed tillering in wheat.

**Figure 5.**
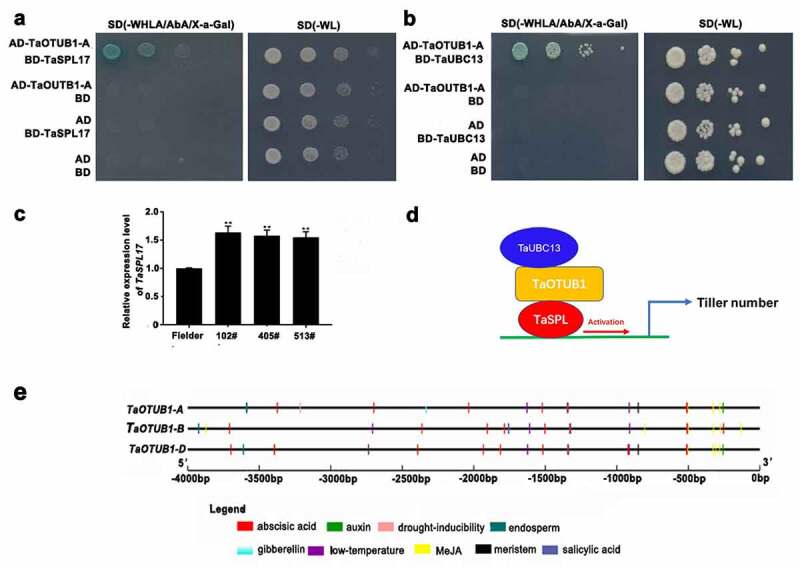
(a) Yeast double hybrid of TaOTUB1-A and TaSPL17. (b) Yeast double hybrid of TaOTUB1-A and TaUBC13. (c) Expression analysis of *TaSPL17* in the wild-type Fielder, 102#, 405#, and 513# plants. Double asterisks indicate significant differences between the wild-type Fielder and *TaOTUB1*-RNAi plants at *P* <0 .01. (d) A proposed working model of the TaOTUB1 in regulating wheat tiller number. (e) Promoter analysis of *TaOTUB1*s.

A tiller is initiated from an axillary bud. We found that TaOTUB1s were mainly expressed in axillary buds. *TaOTUB1*-RNAi transgenic wheat plants showed a reduced tiller number than wild-type plants (Figures 3 and 4). Thus, these results demonstrated that TaOTUB1s were likely involved in regulating the tiller development in wheat. In rice, OTUB1 interacted with the E2 ubiquitin-conjugating protein UBC13 and transcription factor SPL14. The reduced expression or loss-of-function of OTUB1 led to the accumulation of *IPA1*/*SPL14* gene, which resulted in a decrease in the tiller number.^18,19^ *TaSPL17* is the homologous gene of *IPA1/SPL14* in wheat. The yeast double hybrid showed that the TaOTUB1-A protein could interact with TaSPL17 and TaUBC13 proteins in wheat (Figure 5a,b). The qPCR analysis showed that the expression levels of TaOTUB1s decreased while those of *TaSPL17* increased significantly in *TaOTUB1*-RNAi lines (Figure 5c). These results indicated that the OTUB1 pathway might participate in regulating tiller numbers (Figure 5d). It also suggested the mechanisms regulating tiller numbers might be well-conserved in monocots such as rice and wheat. *AtOTU1* is an ortholog of *TaOTUB1, AtOTU1* mutation does not cause visible developmental defects.^22^ It suggests that AtOTU1 does not regulate the branch numbers in Arabidopsis. Therefore, the OTUB1 function in monocots seems to be different from that in dicots.

The plant height, panicle length, and spikelet number of *TaOTUB1*-RNAi transgenic wheat were measured and statistically analyzed to determine whether *TaOTUB1*s affected other agronomic traits of wheat. Unlike in rice, those traits were not significantly changed in the *TaOTUB1*-RNAi transgenic wheat plant, indicating that *TaOTUB1*s might not be involved in regulating these traits (Figure S1). It was probably because the knockdown of *TaOTUB1*s was not effective enough using the RNAi technology. We will investigate the function of *TaOTUB1*s using the CRISPR-Cas9 technology in the future.

### Promoter analysis of *TaOTUB1*s

Hormones and environmental stresses can greatly affect the development and viability of tillers in wheat.^23^ Therefore, the presence of *cis*-regulatory elements in the promoter sequences of all the *TaOTUB1* genes was analyzed. The result showed that these *cis*-regulatory elements included the motifs involved in hormone responses (abscisic acid, auxin, gibberellin, Methyl jasmonate, and salicylic acid) and the binding site involved in drought inducibility and low-temperature stress (Figure 5e). It implied the potential function of *TaOTUB1*s in hormonal regulation and stress tolerance.

## Conclusions

This study was first in reporting the identification, localization analysis, and functional characterization of the wheat *OTUB1* genes. The expressions of *TaOTUB1*s in wheat were involved in developing tillers. The knockdown expression levels of *TaOTUB1s* led to a decreased tiller number similar to that of *OTUB1* in rice. It might be a candidate gene for molecular design breeding in wheat.

## Materials and methods

### Plant materials

For the phenotypic analysis, three T2 *TaOTUB1*-RNAi lines and wild-type Fielder plants were planted in the experimental field of Shandong Agricultural University under natural conditions. All experimental lines were sowed with 25-cm in-row spacing and 15-cm plant-to-plant spacing.

### RNA extraction and qRT-PCR analysis

Total RNA was first extracted from different wheat tissues using TRIzol reagent (Invitrogen, CA, USA) and purified using RNase-free DNaseI (Promega, WI, USA). qRT-PCR analyses were carried out as described previously.^24^ The *TaActin* gene was amplified as an internal control for data normalization. All experiments were independently performed three times under identical conditions. The results were expressed as the mean ± standard errors of three individual experiments. All primers were listed in Supplementary Table S1.

### Subcellular location of TaOTUB1-A protein

For subcellular localization, the full-length cDNA clone of TaOTUB1-A was fused upstream of the GFP gene and inserted into the pROKII vector (stored in our laboratory) to construct a 35S::TaOTUB1-A-GFP vector. Then, the 35S::TaOTUB1-A-GFP vector was transformed into GV3101 *Agrobacterium* strains. The recombinant plasmid was transfected the tobacco leaf for transient expression. Then, the signals were observed using a Leica confocal microscope.

### TaOTUB1-RNAi construct preparation and wheat transformation

The conserved region of TaOTUB1s (the 690- to 960-bp region counted from the translation start site) was inserted into the RNAi vector PC336 to create the *TaOTUB1*-RNAi construct. The PC336 vector was provided by Dr. Daolin Fu (Department of Plant, Soil and Entomological Sciences, University of Idaho, Moscow, Idaho, USA). Then, the *TaOTUB1*-RNAi vector was introduced into the hexaploid wheat (cv Fielder) by *Agrobacterium* infection methods. PCR, herbicide (glufosinate) spraying and a QuickStix Kit for bar were performed to verify the positive transgenic plants.

### Promoter analysis

The 4-kb regions upstream of the start codon of *TaOTUB1*s were considered to be the putative promoter region. The regulatory elements were analyzed using the database of PlantCARE.

### Yeast two-hybrid system

The full coding sequence of *TaOTUB1*-A was inserted into the pGADT7 vector. TaSPL17 and TaUBC13 were cloned and inserted into the pGBKT7 vector. All reconstructed plasmids were sequenced and inserted into yeast strain AH109. Protein interactions were assayed on a selective medium lacking tryptophan, leucine, histidine, and adenine, but supplemented with 20 mg/μL X-α-Gal.

## Supplementary Material

Supplemental Material
