# Supplementary material for: Reducing expression of TaOTUB1s decreases tiller number in wheat
Source: Plant Signal Behav. 2021 Dec 30;16(12):2018217. doi: 10.1080/15592324.2021.2018217 (PMC9208779; doi:10.1080/15592324.2021.2018217)
Supplement: Supplemental Material [file KPSB_A_2018217_SM3526.docx]

| **Primer name** | | **Sequence (5’-3’)** |
| --- | --- | --- |
| qPCR analysis of *TaOUTB1*s | RTQ-TaOTUB1A-F | ACGCACATACCCTTTGTTTC |
|  | RTQ-TaOTUB1A-R | GGTGGGTGACAGGTTGTG |
|  | RTQ-TaOTUB1B-F | GGGCATGAAACTTCTATTGGG |
|  | RTQ-TaOTUB1B-R | CAAAACTGAGCCACGGTCGAAT |
|  | RTQ-TaOTUB1D-F | CAGCCTTACAAGAGGGAACTG |
|  | RTQ-TaOTUB1D-R | AACAAGCAAGAACCACGATTAGTC |
| qPCR analysis of *TaSPL17* | RTQ-TaSPL17-F | ACATCAGCAGCTGCCACATGA |
|  | RTQ-TaSPL17-R | CTACAGAGACCAGTTGGACGAGCT |
| Cloning of *TaOUTB1*s | TaOTUB1-F | CCACAACCTGACGCCCAT |
|  | TaOTUB1-R | TAAAGCATGCTCTGATTA |
| *TaOTUB1-*RNAi construct preparation | GatewayRNAiTaOTUB1-F | CACCTTGCAAGTCTTCTGTGGAG |
|  | GatewayRNAiTaOTUB1-R | TCACTTTGGGTAGAGAATATCA |
| Screening of *TaOTUB1*-RNAi plants | TaOTUB1RNAi-F | CACCTTGCAAGTCTTCTGTGGAG |
|  | TaOTUB1RNAi-R | TCACTTTGGGTAGAGAATATCA |

**Table S1 List of Primers.**
